# Lemon Balm and Corn Silk Extracts Mitigate High-Fat Diet-Induced Obesity in Mice

**DOI:** 10.3390/antiox10122015

**Published:** 2021-12-19

**Authors:** Il-Je Cho, Sung-Eon Kim, Beom-Rak Choi, Hye-Rim Park, Jeong-Eun Park, Seong-Hwa Hong, Young-Sam Kwon, Won-Seok Oh, Sae-Kwang Ku

**Affiliations:** 1 Department of Herbal Prescription, College of Korean Medicine, Daegu Haany University, Gyeongsan 38610, Korea; skek023@dhu.ac.kr; 2 Department of Veterinary Surgery, College of Veterinary Medicine, Kyungpook National University, Daegu 41566, Korea; veter00@knu.ac.kr (S.-E.K.); kwon@knu.ac.kr (Y.-S.K.); 3 Nutracore Co., Ltd., Gwanggyo SK Viewlake A-3206, Beobjo-Ro 25, Suwon 16514, Korea; brchoi@nutracore.co.kr (B.-R.C.); hrpark@nutracore.co.kr (H.-R.P.); jpark@nutracore.co.kr (J.-E.P.); shhong@nutracore.co.kr (S.-H.H.); 4 Department of Veterinary Internal Medicine, College of Veterinary Medicine, Kyungpook National University, Daegu 41566, Korea; 5 Department of Histology and Anatomy, College of Korean Medicine, Daegu Haany University, Gyeongsan 38610, Korea

**Keywords:** corn silk (stigma of *Zea mays* L.), high-fat diet (HFD), lemon balm (*Melisa officinalis* L.), mixture of lemon balm and corn silk extracts (M-LB/CS), obesity

## Abstract

Lemon balm and corn silk are valuable medicinal herbs, which exhibit variety of beneficial effects for human health. The present study explored the anti-obesity effects of a mixture of lemon balm and corn silk extracts (M-LB/CS) by comparison with the effects of single herbal extracts in high-fat diet (HFD)-induced obesity in mice. HFD supplementation for 84 days increased the body weight, the fat mass density, the mean diameter of adipocytes, and the thickness of fat pads. However, oral administration of M-LB/CS significantly alleviated the HFD-mediated weight gain and adipocyte hypertrophy without affecting food consumption. Of the various combination ratios of M-LB/CS tested, the magnitude of the decreases in weight gain and adipocyte hypertrophy by administration of 1:1, 1:2, 2:1, and 4:1 (*w*/*w*) M-LB/CS was more potent than that by single herbal extracts alone. In addition, M-LB/CS reduced the HFD-mediated increases in serum cholesterol, triglyceride, and low-density lipoprotein, prevented the reduction in serum high-density lipoprotein, and facilitated fecal excretion of cholesterol and triglyceride. Moreover, M-LB/CS mitigated the abnormal changes in specific mRNAs associated with lipogenesis and lipolysis in the adipose tissue. Furthermore, M-LB/CS reduced lipid peroxidation by inhibiting the HFD-mediated reduction in glutathione, catalase, and superoxide dismutase. Therefore, M-LB/CS is a promising herbal mixture for preventing obesity.

## 1. Introduction

Obesity is the abnormal accumulation of body fat and exaggerates the incidence of comorbidities, including cardiovascular disease, diabetes mellitus, non-alcoholic fatty liver disease, and neurodegenerative disease [1]. In 2016, it has been estimated that about 39% of adults worldwide are overweight (i.e., body mass index ≥ 25) and 13% are obese (i.e., body mass index ≥ 30) [2]. In East Asian countries, including Korea, the prevalence of obesity within last two decades has been rapidly escalated due to excess calorie intake and sedentary lifestyles along with socioeconomic growth. Although approved pharmacologic interventions (e.g., orlistat, phentermine/topiramate, naltrexone/bupropion, and liraglutide) as well as changes in lifestyle (e.g., restricted and balanced food intake with exercise) have been recommended to manage obesity [3,4], weight control is still extremely challenging to achieve and sustain. In addition, anti-obesity drugs have been frequently discontinued and under-prescribed, owing to their unacceptable side effects. In this regard, edible medicinal herbs with fewer side effects have been receiving great attention as a complementary and alternative approach for long-term management of obesity. 

The pharmacological activities of medicinal herbs have been believed to be due to the flavonoids contained in the parental herbs [5]. Flavonoids are ubiquitous polyphenolic phytochemicals in medicinal plants, and about 4000 flavonoids have been isolated from medicinal herbs. Since oxidative stress and chronic low-grade inflammation have been considered as pivotal mechanisms in the pathogenesis of obesity [3,6], various flavonoids with potent antioxidant and anti-inflammatory activities have been reported to be promising candidates for the treatment or prevention of obesity [6,7].

Among diverse medicinal herbs enriched with flavonoids, lemon balm (*Melissa officinalis* L.) is a fragrant herb that has been ingested for remedying mental, cardiovascular, and respiratory disorders in traditional medicine [8]. In addition, corn silk, which is thread-like in style and is a stigma of *Zea mays* L., has been traditionally used to treat many complications, including nephritis, cystitis, and edema [9]. Modern pharmacological studies using lemon balm and corn silk have been identified a variety of bioactive phytochemicals (e.g., flavonoids, terpenoids, and alkaloids) responsible for their beneficial effects, such as antioxidant and anti-inflammatory activities [8,10]. Moreover, studies have suggested that lemon balm and corn silk are potential herbs for preventing obesity [11,12,13,14,15,16]. Furthermore, throughout preliminary screening study from our laboratory, we also found that lemon balm and corn silk extracts could suppress weight gain in experimental animals fed a high-fat diet (HFD) (data not shown). However, further research is needed on the enhanced anti-obesity effects through combinations of two herbs.

As part of the process to develop valuable herbal mixtures, which can promote human health, the following strategies have been used to minimize the number of animals required: (1) screening of individual herbal extracts, (2) optimization of combination ratio, and (3) dose–response experiments of herbal mixtures. Throughout these strategies, we recently tested various combination ratios of 1:8 to 8:1 of lemon balm and dandelion mixtures as a second step and discovered a potent hepatoprotective herbal mixture containing lemon balm and dandelion extracts in a ratio of 2:1 (*w*/*w*) [17,18]. Therefore, the aim of the present study was to explore the efficacy of various combination ratios of mixtures of lemon balm/corn silk (M-LB/CS) in HFD-induced obese mice and to elucidate the optimal combination ratio by comparing prophylactic effects of M-LB/CS with single herbal extract alone.

## 2. Materials and Methods

### 2.1. Quantification of Rosmarinic Acid and Allantoin by High-Performance Liquid Chromatography (HPLC)

Lemon balm hydroethanolic extract (LBE) was supplied from Evear Extraction (Coutures, France), and liquid concentrate of corn silk, provided by Xi’an Sost Biological Sciences and Technology Co., Ltd. (Xi’an, China), was evaporated to obtain corn silk extract (CSE). After LBE and rosmarinic acid (Sigma-Aldrich, St. Louis, MO, USA) were dissolved in 50% methanol, samples were eluted in Zorbax Eclipse C_18_ column (250 × 4.6 mm, 5 μm pore size; Agilent Technologies, Palo Alto, CA, USA) with a mobile phase comprising 1:3 acetonitrile and acetic acid, respectively. Eluants were detected at 330 nm, and concentration of rosmarinic acid in LBE was calculated by interpolating the peak area with the same retention time as the standard curve of rosmarinic acid. For quantifying allantoin level in CSE, CSE and allantoin (Sigma-Aldrich) were dissolved in distilled water and then eluted using an Asahipak NH2P-50 column (250 × 4.6 mm, 5 μm pore size; Shodex, Tokyo, Japan). Gradient solution from 15% to 30% acetonitrile was used for separating compounds in CSE, and eluants were monitored at 200 nm.

### 2.2. Animal Husbandry and Treatment

After experimental protocol using animals was approved by the Institutional Animal Care and Use Committee of Daegu Haany University (Approval No., DHU2019-002), 104 SPF/VAF CrljOri:CD1 [ICR] mice (age—6 weeks old; gender—female), obtained from OrientBio (Seungnam, Gyeonggi-do, Korea), were acclimatized for 7 days (temperature—20–25 °C; humidity—40–45%; 12:12 h of light:dark cycle) with standard rodent chow (Purinafeed; Seungnam, Gyeonggi-do, Korea) and water. For adapting mice to the experimental diet, all but 8 mice (*n* = 96) were fed an HFD, which provides 45% of the energy from fat (Research Diet; New Brunswick, NJ, USA), for 7 days. Meanwhile, the remaining mice (*n* = 8) were fed a normal fat diet (NFD) (Purinafeed). After, the mice fed an HFD were randomly divided into twelve groups (*n* = 8 mice per group), fed and indicated herein as follows: a group of mice fed an HFD only—HFD; group administered LBE with HFD—HFD + LBE; group administered CSE with HFD—HFD + CSE; group administered 1:1 mixture (*w*/*w*) of LBE and CSE with HFD—HFD + M-LB/CS (1:1); group administered 1:2 mixture (*w*/*w*) of LBE and CSE with HFD—HFD + M-LB/CS (1:2); group administered 1:4 mixture (*w*/*w*) of LBE and CSE with HFD—HFD + M-LB/CS (1:4); group administered 1:6 mixture (*w*/*w*) of LBE and CSE with HFD—HFD + M-LB/CS (1:6); group administered 1:8 mixture (*w*/*w*) of LBE and CSE with HFD—HFD + M-LB/CS (1:8); group administered 2:1 mixture (*w*/*w*) of LBE and CSE with HFD—HFD + M-LB/CS (2:1); group administered 4:1 mixture (*w*/*w*) of LBE and CSE with HFD—HFD + M-LB/CS (4:1); group administered 6:1 mixture (*w*/*w*) of LBE and CSE with HFD—HFD + M-LB/CS (6:1); group administered 8:1 mixture (*w*/*w*) of LBE and CSE with HFD—HFD + M-LB/CS (8:1). LBE, CSE, or various combination ratios of M-LB/CS (200 mg/kg each), dissolved in distilled water, were orally administered into the mice once a day for 84 days (i.e., day 0 = the first day administered herbal extract). In the case of the mice fed with only NFD or HFD, an equivalent volume of distilled water was orally administered instead of treating with the herbal extract. All the mice were euthanized on day 84 (i.e., 24 h after the last administration of herbal extract), and fat tissues and bloods were collected for the subsequent experiments.

### 2.3. Measurement of Body and Fat Weight

On day 0 and 84, all mice were fasted overnight to minimize the differences from feeding. Changes in body weight were measured on day 0, 1, 7, 14, 21, 28, 35, 42, 49, 56, 63, 70, 77, 83, and 84 using a balance (Precisa Instruments, Zürich, Switzerland). After measuring the weights of left periovarian fat fad and abdominal fat fad attached to the quadratus lumborum muscle, the relative weight of fat pad was calculated as a percentage of the body weight on day 84.

### 2.4. Measurement of Mean Daily Food Consumption

Daily food consumption was calculated by weighing the remaining diet 24 h after feeding and dividing by the number of mice in each cage. During the experimental period, food intake was measured once a week to calculate the mean daily food consumption.

### 2.5. Measurement of Body and Abdominal Fat Density

To analyze body and abdominal fat density, euthanized mice were scanned using a dual-energy X-ray absorptiometry (DEXA) (InAlyzer; Medikors, Seungnam, Korea), as previously described [19,20].

### 2.6. Histological Analyses

Fixation of abdominal and periovarian fat pads, paraffin embedding, sectioning, and hematoxylin and eosin staining were conducted, as described previously [19,20]. After observing fat tissues under light microscope (Eclipse 80*i*; Nikon, Tokyo, Japan), the thickness of the deposited fat pad (mm) and the mean diameter of 10 adipocytes (μm) were measured using an automated image analyzer (*i*Solution FL ver9.1; IMT *i*-solution Inc., Burnaby, BC, Canada).

### 2.7. Measurement of Lipid Profiles

After separating serum from whole blood, serum levels of cholesterol, triglyceride, low-density lipoprotein (LDL), and high-density lipoprotein (HDL) were determined using an automated blood analyzer (Dri-Chem NX500i; Fuji Medical System, Tokyo, Japan). In addition, lipids were extracted from feces that had been collected 8 h after the last herbal extract administration. Fecal levels of cholesterol and triglyceride were calorimetrically measured using total cholesterol assay kit (Cell Biolabs, San Diego, CA, USA) and a triglyceride assay kit (Cayman, Ann Arbor, MI, USA) according to the manufacturer’s instructions.

### 2.8. Quantitative Polymerase Chain Reaction (qPCR)

Total RNAs were extracted from the periovarian fat tissue using Trizol reagent (Invitrogen, Carlsbad, CA, USA) and DNase I (Ambion, Austin, TX, USA), and then reverse-transcribed using a High-Capacity cDNA Reverse Transcription Kit (Applied Biosystems, Foster City, CA, USA). Specific genes associated with lipid metabolism were amplified using StepOnePlus^TM^ Real-Time PCR System (Applied Biosystems). Primer sequences are listed in Table 1. Relative expression of specific genes was quantified based on comparative C_T_ method [21], and glyceraldehyde 3-phosphate dehydrogenase (GAPDH) was used as a housekeeping gene.

### 2.9. Measurement of Lipid Peroxidation and Antioxidant Activities

Lipid peroxidation, reduced glutathione level, and specific activities of catalase and superoxide dismutase were determined using liver homogenates, according to the previously established methods [19,20]. 

### 2.10. Statistical Analysis

All values are expressed as the mean ± standard deviation of eight mice. SPSS Statistics for Windows (Release 14.0 K; SPSS Inc., Chicago, IL, USA) was used for comparing the means among experimental groups. Samples showing equal variance were analyzed by analysis of variance test, followed by the least significant differences. On the other hand, samples with heterogeneity of variance were analyzed by Mann–Whitney U test after conducting Kruskal–Wallis H test. *p* < 0.05 was considered as statistical significance.

## 3. Results

### 3.1. Mixture of Lemon Balm and Corn Silk Extracts Reduces Body Weight Gain in Mice Fed an HFD

**Figure 1:**
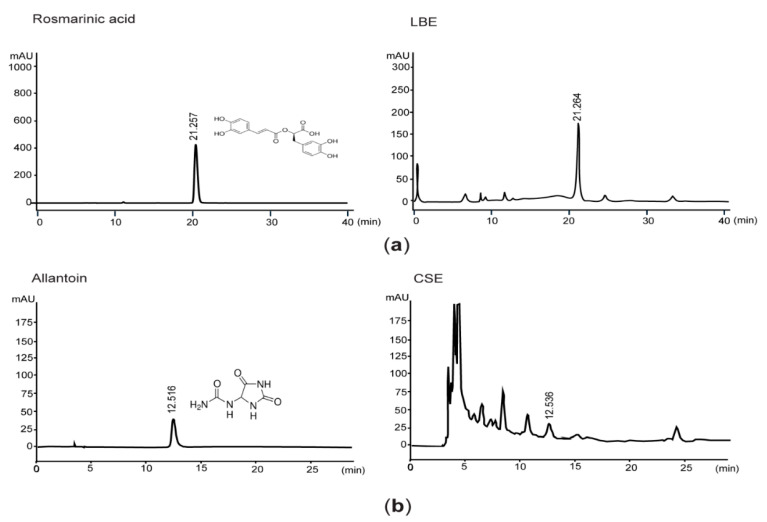
Quantification of rosmarinic acid and allantoin in lemon balm extract (LBE) and corn silk extract (CSE). (**a**) Eluants after loading rosmarinic acid (**left**) and LBE (**right**) were detected at the wavelength of 330 nm. (**b**) Eluants of allantoin (**left**) and CSE (**right**) were detected at 200 nm. The numbers in each chromatogram are retention times (min).

Because rosmarinic acid and allantoin have been known as bioactive phytochemicals found in LBE and CSE [17,22], we quantified the concentrations of rosmarinic acid and allantoin in LBE and CSE. Results from HPLC analyses indicated that the LBE and CSE used in the present study contained 46.01 ± 9.20 mg/g of rosmarinic acid and 1.60 ± 0.32 mg/g of allantoin, respectively (Figure 1).

**Figure 2:**
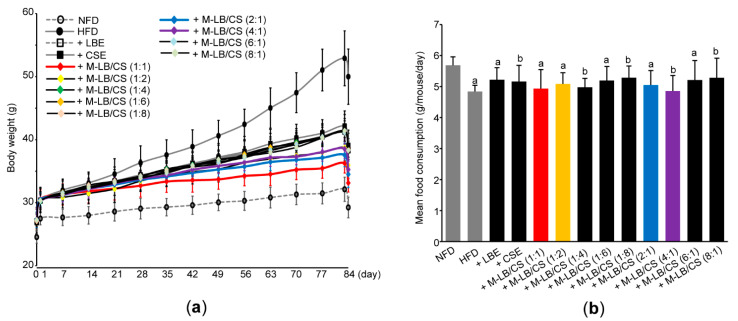
Mixture of lemon balm and corn silk extracts (M-LB/CS) reduced body weight gain in mice fed a high-fat diet (HFD). (**a**) Changes in body weight during the experimental period. Mice were fed an NFD or HFD and administered with LBE, CSE, or various combination ratios of M-LB/CS, as described in the Materials and Methods Section. All mice were fasted for 12 h on days 0 and 84. (**b**) Mean daily food consumption. ^a^ *p* < 0.01, ^b^ *p* < 0.05, vs. NFD; NFD—normal fat diet.

Next, we adopted an HFD-induced obesity model to investigate the anti-obesity potential of M-LB/CS in vivo. For this purpose, ICR mice (*n* = 96) were fed a 45%/kcal HFD. A period of 7 days of supplementation of an HFD significantly increased the body weight as compared with NFD (i.e., 27.40 ± 0.87 g in mice fed an NFD vs. 30.25 ± 1.34 g in mice fed an HFD). After 1 week adaptation period, mice fed an HFD were randomly assigned to twelve groups (e.g., HFD, HFD + LBE, HFD + CSE, HFD + M-LB/CS (1:1), HFD + M-LB/CS (1:2), HFD + M-LB/CS (1:4), HFD + M-LB/CS (1:6), HFD + M-LB/CS (1:8), HFD + M-LB/CS (2:1), HFD + M-B/CS (6:1), and HFD + M-LB/CS (8:1)) and administered LBE, CSE, or various combination ratios of M-LB/CS (200 mg/kg each) once a day for 84 days. On day 0 (i.e., the first day administered herbal extract), there were no differences in body weight among experimental groups, except mice fed an NFD. However, as compared with the HFD group, a significant reduction in body weight was shown from 14 days after administration of M-LB/CS (1:2); 21 days after administration of M-LB/CS (1:1), M-LB/CS (1:6), and M-LB/CS (2:1); 28 days after administration of M-LB/CS (4:1), M-LB/CS (6:1), and M-LB/CS (8:1); and 35 days after administration of M-LB/CS (1:4), M-LB/CS (1:8), LBE and CSE, respectively (Figure 2a). During 84 days of the experimental period, body weight gains in NFD-, HFD-, HFD + LBE-, HFD + CSE-, HFD + M-LB/CS (1:1)-, HFD + M-LB/CS (1:2)-, HFD + M-LB/CS (1:4)-, HFD + M-LB/CS (1:6)-, HFD + M-LB/CS (1:8)-, HFD + M-LB/CS (2:1)-, HFD + M-LB/CS (4:1)-, HFD + M-LB/CS (6:1)-, and HFD + M-LB/CS (8:1)-administered mice were 4.70 ± 1.26, 23.01 ± 4.43, 11.24 ± 1.41, 12.19 ± 1.43, 5.78 ± 1.63, 8.86 ± 1.51, 11.01 ± 2.20, 11.38 ± 2.64, 11.30 ± 1.75, 7.15 ± 1.36, 8.03 ± 1.58, 11.06 ± 1.34, and 11.03 ± 1.31 g, respectively. Administration of herbal extract significantly reduced the HFD-mediated body weight gain. In particular, the magnitude of the decrease in body weight gain by M-LB/CS (1:1), M-LB/CS (1:2), M-LB/CS (2:1), and M-LB/CS (4:1) was potent as compared with a single herbal extract alone. Although the HFD slightly, but significantly, decreased mean daily food consumption as compared with NFD, there were no differences in mean daily food consumption among experimental groups administered with herbal extracts (Figure 2b).

### 3.2. Mixture of Lemon Balm and Corn Silk Extracts Decreases Fat Deposition in Mice Fed an HFD

**Figure 3:**
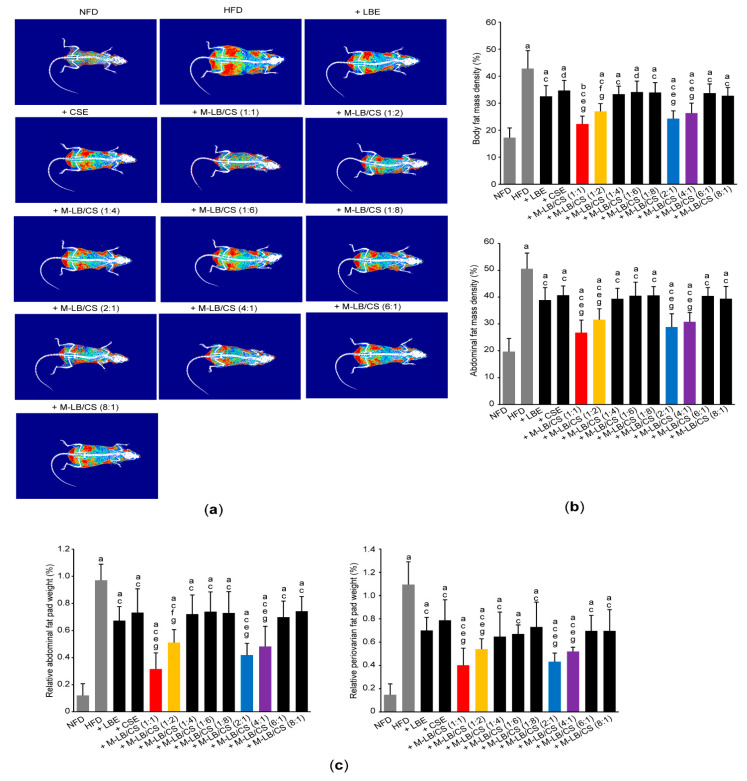
M-LB/CS decreases fat deposition in mice fed an HFD. (**a**) Representative DEXA images of experimental groups. (**b**) Fat mass densities of whole body (**upper**) and abdominal region (**lower**) were calculated from DEXA. (**c**) Abdominal (**left**) and periovarian (**right**) fat pad weights were normalized by body weight. ^a^ *p* < 0.01, ^b^ *p* < 0.05, vs. NFD; ^c^ *p* < 0.01, ^d^ *p* < 0.05, vs. HFD; ^e^ *p* < 0.01, ^f^ *p* < 0.05, vs. HFD + LBE; ^g^ *p* < 0.01, vs. HFD + CSE; DEXA—dual-energy X-ray absorptiometry.

To explore whether M-LB/CS decreases HFD-induced body weight gain via modulating fat deposition, we scanned fat density of the entire body and the abdominal region using a DEXA (Figure 3a). As expected, significant increases in fat density of the entire body and the abdominal region were shown in group of mice fed an HFD. However, the HFD-mediated increases in fat deposition were significantly prevented by administrating various combination ratios of M-LB/CS. In addition, the magnitude of the decreases in fat density, mediated by M-LB/CS (1:1), M-LB/CS (1:2), M-LB/CS (2:1), and M-LB/CS (4:1) was greater than that by single herbal extract alone (Figure 3b). Similarly, administration of M-LB/CS (1:1), M-LB/CS (1:2), M-LB/CS (2:1), and M-LB/CS (4:1) significantly blocked the HFD-mediated increases in the relative weight of abdominal and periovarian fat pad, as compared with a single herbal extract alone (Figure 3c).

**Figure 4:**
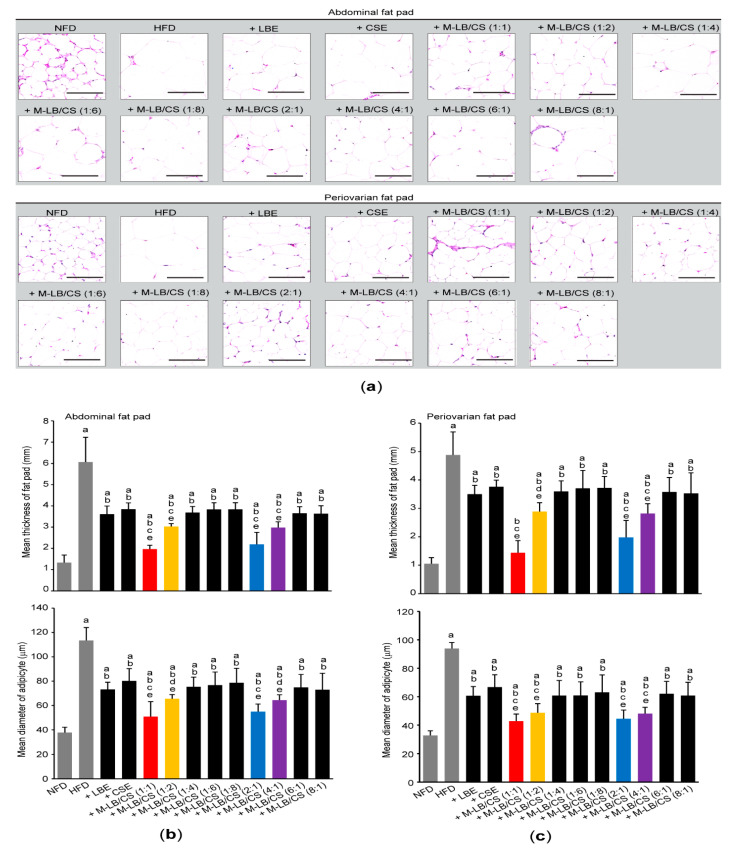
M-LB/CS reduces the hypertrophy of adipose tissue. Abdominal and periovarian fat pads were stained with hematoxylin and eosin (**a**). Thickness of fat fad (**upper**) and mean diameter of adipocytes (**lower**) from abdominal (**b**) and periovarian (**c**) fat pad were calculated using an image analyzer. ^a^ *p* < 0.01, vs. NFD; ^b^ *p* < 0.01, vs. HFD; ^c^ *p* < 0.01, ^d^ *p* < 0.05, vs. HFD + LBE; ^e^ *p* < 0.01, vs. HFD + CSE.

To further investigate the effect of M-LB/CS administration on HFD-mediated histopathological changes in mice, tissue sections, prepared from the abdominal region, as well as periovarian fat pads were stained with hematoxylin and eosin (Figure 4a). Histomorphometric analyses against the abdominal fat pads indicated that an HFD significantly increased the thickness of fat fad and the mean diameter of adipocytes. However, administration of various combination ratios of M-LB/CS significantly alleviated the hypertrophic changes of abdominal fat pad in response to HFD. In particular, the magnitude of decreases in the fat pad thickness and adipocyte’s mean diameter, caused by M-LB/CS (1:1), M-LB/CS (1:2), M-LB/CS (2:1), and M-LB/CS (4:1) administration, was greater than that by single herbal extract alone (Figure 4b). In parallel with the results obtained from abdominal fat pad, M-LB/CS also significantly blocked the HFD-mediated hypertrophy of the periovarian fat pad, and the reducing effects by 1:1, 1:2, 2:1, and 4:1 combination ratios of M-LB/CS were more potent than those by the single herb (Figure 4c).

### 3.3. Mixture of Lemon Balm and Corn Silk Extracts Prevents Impaired Lipid Profiles in Mice Fed an HFD

**Figure 5:**
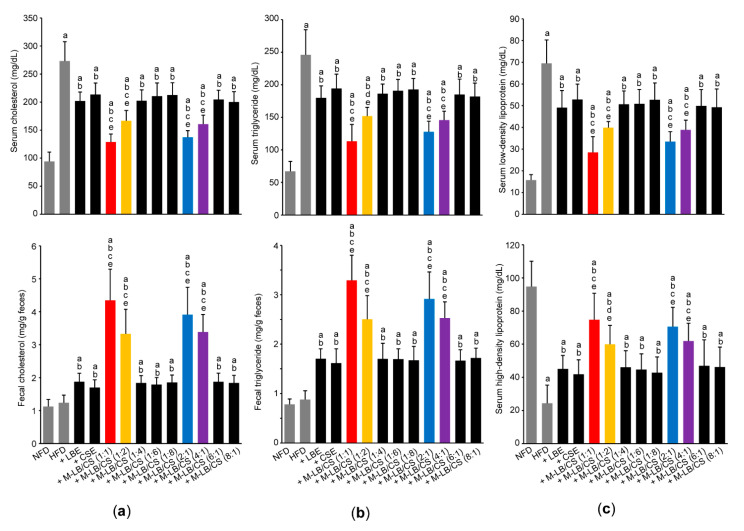
M-LB/CS prevents impaired lipid profiles. Serum (**upper**) and fecal (**lower**) cholesterol (**a**), serum and fecal triglycerides (**b**), and serum low- and high-density lipoprotein (**c**) were determined. ^a^ *p* < 0.01, vs. NFD; ^b^ *p* < 0.01, vs. HFD; ^c^ *p* < 0.01, ^d^ *p* < 0.05, vs. HFD + LBE; ^e^ *p* < 0.01, vs. HFD + CSE.

Next, lipid profiles in the serum and feces were observed to investigate whether M-LB/CS administration can reduce abnormal lipids distribution induced by HFD. As expected, the HFD significantly increased the serum levels of cholesterol, triglyceride, and LDL (Figure 5a—upper; Figure 5b—upper; and Figure 5c—upper). In addition, HDL in serum was significantly decreased in mice fed an HFD (Figure 5c—lower). However, these abnormal changes in the serum lipid levels were significantly mitigated in mice administered with various combination ratios of M-LB/CS. Indeed, preventive effects of serum lipid profiles in mice administered with M-LB/CS (1:1), M-LB/CS (1:2), M-LB/CS (2:1), and M-LB/CS (4:1) were more potent that those by single herbal extract alone (Figure 5a—upper; Figure 5b—upper; and Figure 5c). Although HFD supplementation did not change the fecal level of cholesterol and triglyceride, M-LB/CS significantly increased the fecal excretion of cholesterol and triglyceride. In particular, the magnitude of fecal lipids excretion in mice administered with 1:1, 1:2, 2:1, and 4:1 combination ratios of M-LB/CS was greater than that by single herb (Figure 5a—lower and Figure 5b—lower).

### 3.4. Mixture of Lemon Balm and Corn Silk Extracts Mitigates Abnormal Changes in Specific Genes Associated with Lipid Metabolism in the Adipose Tissue

To investigate whether M-LB/CS prevents the diet-induced obesity through modulating lipid metabolism in the adipose tissue, qPCR analysis was conducted (Table 2). As expected, HFD significantly increased mRNA levels of C/EBPα, C/EBPβ, PPARγ, and SREBP-1c, which are representative genes associated with lipogenesis and adipocyte differentiation [23,24]. However, various combination ratios of M-LB/CS significantly blocked the induction of C/EBPα, C/EBPβ, PPARγ, and SREBP-1c. The magnitude of the reductions by 1:1, 1:2, 2:1, and 4:1 combination ratios of M-LB/CS was greater than that caused by single herbal extract alone. On the contrary, HFD significantly inhibited specific mRNAs involved in lipolysis and energy expenditure (e.g., PPARα and UCP2) [23,25]. Moreover, reciprocal changes on mRNA expression of leptin and adiponectin were shown in mice fed an HFD. However, M-LB/CS administration significantly prevented the changes in PPARα, UCP2, leptin, and adiponectin mRNAs caused by HFD. Furthermore, the inhibitory effects in mice administered with 1:1, 1:2, 2:1, and 4:1 combination ratios of M-LB/CS were more potent than those of single herbal extract alone.

### 3.5. Mixture of Lemon Balm and Corn Silk Extracts Scavenges Lipid Peroxidation by Recovering Antioxidant Activities

**Figure 6:**
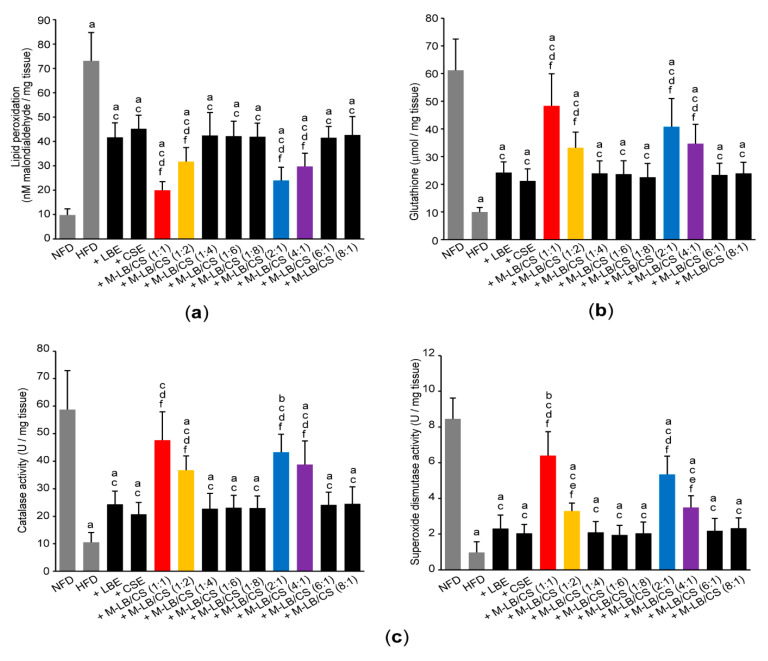
M-LB/CS scavenges lipid peroxidation. Malondialdehyde (**a**) and reduced glutathione (**b**) levels and specific activities of catalase and superoxide dismutase (**c**) were measured using liver homogenates. ^a^ *p* < 0.01, ^b^ *p* < 0.05, vs. NFD; ^c^ *p* < 0.01, vs. HFD; ^d^ *p* < 0.01, ^e^ *p* < 0.05, vs. HFD + LBE; ^f^ *p* < 0.01, vs. HFD + CSE.

To explore whether M-LB/CS can restore antioxidant activities by decreasing abnormal lipids accumulation, we next determined lipid peroxidation by measuring concentration of malondialdehyde in the hepatic tissue. Administration of various combination ratios of M-LB/CS significantly suppressed the increase in lipid peroxidation caused by HFD supplementation. In addition, the magnitude of the reduction in lipid peroxidation in mice administered with 1:1, 1:2, 2:1, and 4:1 (*w*:*w*) M-LB/CS was potent, as compared with mice administered with single herbal extract alone (Figure 6a). In conjunction with the increase in lipid peroxidation, HFD significantly decreased the level of reduced glutathione (an endogenous antioxidant) and specific activities of catalase and superoxide dismutase (representative antioxidant enzymes). However, HFD-mediated decreases in antioxidant activities were significantly prevented by M-LB/CS administration, and the magnitude of the preventive effects by 1:1, 1:2, 2:1, and 4:1 (*w*:*w*) M-LB/CS was greater than that by single herbal extract alone (Figure 6b,c).

## 4. Discussion

For controlling of the quality of M-LB/CS, in the present study, rosmarinic acid and allantoin were quantified as enriched phytochemicals found in LBE and CSE, respectively. Our supplementary in vitro results indicated that rosmarinic acid could scavenge 2,2-Diphenyl-1-picrylhydrazyl (DPPH) radical in a concentration-dependent manner (IC_50_, 2.25 ± 0.03 μg/mL). However, allantoin, up to 300 μg/mL, did not remove the radical produced from DPPH (Figure S1), which are parallel with previous observation that rosmarinic acid and allantoin exhibit (in)direct antioxidant activities [26,27,28]. Rosmarinic acid is a derivative of hydroxycinnamic acid in which caffeic acid and 3,4-dihydroxyphenyllactic acid are linked by an ester bond. Accumulated studies suggest that rosmarinic acid possesses a number of beneficial activities for promoting human health. For instance, rosmarinic acid inhibits the progression of diverse stage of liver diseases, reduces neurogenic and inflammatory pains, and leads apoptosis of various cancer cells [29,30,31,32]. In addition, in vitro studies using adipocytes indicated that rosmarinic acid can inhibit differentiation of preadipocytes via reducing adipogenesis and suppressing inflammation [33,34]. On the other hand, allantoin is a purine-derived phytochemical that is frequently found in *Z. mays*, *Dioscorea rhizome*, *Nelumbo nucifera*, and some leguminous plants [35,36,37,38]. Allantoin has been used as a representative ingredient in astringent and keratolytic cosmetics because it stimulates wound healing through promoting the proliferation of fibroblasts [39]. In addition, allantoin has been reported to reduce airway inflammation, improve cognitive function, and protect the gastric tissue [35,40,41]. In particular, intraperitoneal injection of allantoin can alleviate HFD-induced obesity and hyperlipidemia, and those effects are abolished by efaroxan pretreatment [36,42], which provides evidence that the activation of the imidazoline I_1_-receptor is associated with allantoin-mediated maintenance of lipid homeostasis. Although rosmarinic acid (10–200 mg/kg) and allantoin (5 mg/kg) have been reported to possess beneficial effects in mice fed an HFD [36,43,44,45], the effective concentrations of rosmarinic acid and allantoin administered directly into animals in the aforementioned references appears too high to be reached in vivo by administration of 200 mg/kg M-LB/CS in the present study. In addition, HPLC chromatogram after eluting LBE and CSE indicated that there were many peaks beyond rosmarinic acid and allantoin, and several phytochemicals contained in two herbs (e.g., ursolic acid, oleanolic acid, caffeic acid, luteolin, rutin, maysin, and α-terpineol) also have potential to alleviate obesity [46,47,48,49,50,51,52]. Therefore, not only rosmarinic acid/allantoin, but also other phytochemicals existing in LBE and CSE may collaboratively contribute to reduce hypertrophy of the fat tissue and resolve abnormal lipid metabolism.

Owing to the gender differences in food consumption, physical activity, and so on, prevalence of obesity in females is generally higher than in males [53]. In this regard, we previously established HFD-induced obesity model using female ICR mice [19,20,54]. To elucidate optimal ratio of M-LB/CS showing anti-obesity activity, female mice were administered HFD and various combination ratios of M-LB/CS for 84 days. As compared with the mice group administered with single herbal extract alone, the present study showed that 1:1, 1:2, 2:1, and 4:1 ratios of M-LB/CS decreased body weight gain and fat mass density. In addition, histomorphometric analyses indicated that reduction in the body weight by M-LB/CS resulted from mitigating hypertrophy of the adipocytes.

In the present study, we showed that abnormal lipid profiles in the serum induced by HFD were alleviated by M-LB/CS. In particular, M-LB/CS significantly increased the fecal excretion of cholesterol and triglyceride. It has been reported that black tea polyphenols, oolong tea extract, celastrol, montmorillonite, and orlistat can attenuate diet-induced obesity via accelerating fecal excretion of lipids [3,55,56,57,58]. Inhibition of pancreatic lipase, downregulation of intestinal transporter for fatty acids and cholesterols, and alteration of intestinal microbiota have been suggested as possible mechanisms for enhancing fecal lipid excretion via inhibition of lipid absorption [3,55,59]. Although detailed mechanisms for M-LB/CS must be further established, the increase in fecal lipids excretion by M-LB/CS will serve one of the plausible mechanisms to explain the anti-obesity activity of M-LB/CS through alleviating the abnormal accumulation of lipids in the blood that occurs in HFD-induced obesity in mice.

PPARs belong type II nuclear receptor superfamily and are transcription factors, which govern nutrient and energy metabolisms after binding of lipophilic ligands [23]. Three PPAR subtypes (i.e., PPARα, PPARβ/δ, and PPARγ) share structural similarity, but differ in pathophysiological function via inducing different set of genes. For instance, PPARα, in the adipose tissue, promotes lipolysis by regulating essential genes correlated with triglyceride hydrolysis (e.g., lipase), fatty acid uptake (e.g., cluster of differentiation 36 and carnitine palmitoyltransferase), β-oxidation (e.g., acyl-CoA oxidase), and thermogenesis (e.g., UCP) [23,25]. On the contrary, PPARγ facilitates preadipocytes maturation (e.g., C/EBPs) and lipogenesis (e.g., SREBP-1) in the adipose tissue [23,24]. Interestingly, studies from in vitro ligand binding assay and reporter gene assay, harboring PPAR response element, indicated that LBE is capable of binding and transactivating three canonical PPARs [12,60]. In addition, it has been reported that the inhibition of triglyceride accumulation by treatment of lemon balm extract was blocked by pretreatment with GW6471 (a PPARα antagonist) [12]. Therefore, present results showing 1:1, 1:2, 2:1, and 4:1 ratios of M-LB/CS prevented the altered expression of PPARs mRNA in response to HFD imply that M-LB/CS may regulate adipocyte hypertrophy and obesity primarily through modulating PPARs. Furthermore, the results by M-LB/CS provide the possibility that not only LBE but also CSE may also contain several phytochemicals for transactivating PPARs. Further research will be necessary to elucidate the responsible phytochemicals in M-LB/CS.

In parallel with previous reports that HFD-enlarged adipocytes secrete more leptin and less adiponectin [15,61], the present study also showed an increase in leptin mRNA and a decrease in adiponectin mRNA in the fat tissues obtained from the mice fed an HFD. In the adipose tissue, adiponectin encourages the ‘healthy expansion of adipocytes’ via stimulating adipogenesis and storing fats from other peripheral tissues, while maintaining insulin sensitivity [62]. Thus, the inhibition of an HFD-dependent decrease in adiponectin mRNA by M-LB/CS suggests that M-LB/CS may facilitate systemic energy homeostasis by recovering adiponectin mRNA. In addition, because leptin has been considered as a critical adipokine to regulate appetite in the hypothalamus [62,63], the present result, showing the reduction in mean daily food consumption by HFD, might be due to an increased production of leptin in the adipose tissue. Significantly, we showed that administration of M-LB/CS did not change the reduction in food consumption by HFD, while M-LB/CS significantly decreased the mRNA level of leptin induced by HFD. Discrepancy between leptin mRNA and food consumption by M-LB/CS suggests that M-LB/CS may attenuate obesity via activating peripheral lipid catabolism (e.g., restoration of adiponectin mRNA and modulation of PPARs) without affecting central leptin resistance.

## 5. Conclusions

In conclusion, the present study showed that 1:1, 1:2, 2:1, and 4:1 ratios of M-LB/CS reduced body weight in HFD-induced obesity in female mice by inhibiting hypertrophy of adipocytes and abnormal fat deposition. In addition, M-LB/CS prevented abnormal changes in serum lipid profiles via promoting fecal lipids excretion. Finally, M-LB/CS not only restored the altered expression of specific genes involved in lipid metabolism, but also abrogated lipid peroxidation by recovering antioxidant activities. The anti-obesity effects by M-LB/CS (1:1) administration were the most potent among the various combination ratios tested. If further appropriate procedures (e.g., dosage-dependent efficacy experiments, drug interaction study by herbal combination, toxicological study, and clinical trial for human application) for M-LB/CS are successfully conducted, M-LB/CS will be a promising nutraceutical for preventing obesity or obesity-mediated metabolic disorders.

## Figures and Tables

**Table 1:** Primer sequences used in the present study.

Gene Name	Forward Primer	Backward Primer	RefSeq Accession No.	Amplicon Size (bp)
Adiponectin	5′-CCCAAGGGAACTTGTGCAGGTTGGATG-3′	5′-GTTGGTATCATGGTAGAGAAGAAAGCC-3′	NM_009605.5	639
C/EBPα	5′-TGGACAAGAACAGCAACGAGTAC-3′	5′-CGGTCATTGTCACTGGTCAACT-3′	NM_001287514.1	136
C/EBPβ	5′-AAGCTGAGCGACGAGTACAAGA-3′	5′-GTCAGCTCCAGCACCTTGTG-3′	NM_001287738.1	116
GAPDH	5′-CATCTTCCAGGAGCGAGACC-3′	5′-TCCACCACCCTGTTGCTGTA-3′	NM_001289726.1	753
Leptin	5′-CCAAAACCCTCATCAAGACC-3′	5′-GTCCAACTGTTGAAGAATGTCCC-3′	NM_008493.3	390
PPARα	5′-ATGCCAGTACTGCCGTTTTC-3′	5′-GGCCTTGACCTTGTTCATGT-3′	NM_011144.6	220
PPARγ	5′-AGTGGAGACCGCCCAGG-3′	5′-GCAGCAGGTTGTCTTGGATGT-3′	NM_00127330.2	64
SREBP-1c	5′-AGCCTGGCCATCTGTGAGAA-3′	5′-CAGACTGGTACGGGCCACAA-3′	NM_011480.4	132
UCP2	5′-CCGCATTGGCCTCTACGACTCT-3′	5′-CCCCGAAGGCAGAAGTGAAGTG-3′	NM_011671.5	386

C/EBP—CC/AAT enhancer binding proteins; GAPDH—glyceraldehyde 3-phosphate dehydrogenase; PPAR—peroxisome proliferator-activated receptor; SREBP—sterol regulatory element binding protein; UCP—uncoupling protein.

**Table 2:** M-LB/CS alleviates impaired mRNA expression associated with lipid metabolism.

Experimental Group	Relative mRNA Level (Folds)							
	C/BEPα	C/BEPβ	PPARγ	SREBP-1c	PPARα	UCP2	Leptin	Adiponectin
NFD	1.00 ± 0.06	1.01 ± 0.06	1.00 ± 0.07	1.01 ± 0.06	1.00 ± 0.04	1.02 ± 0.08	1.03 ± 0.08	0.99 ± 0.16
HFD	2.05 ± 0.34 ^a^	3.74 ± 0.86 ^a^	7.17 ± 1.01 ^a^	2.46 ± 0.54 ^a^	0.22 ± 0.05 ^a^	0.20 ± 0.06 ^a^	6.94 ± 0.99 ^a^	0.14 ± 0.07 ^a^
HFD + LBE	1.53 ± 0.07 ^a,b^	2.48 ± 0.41 ^a,b^	4.79 ± 0.71 ^a,b^	1.75 ± 0.15 ^a,b^	0.36 ± 0.08 ^a,b^	0.36 ± 0.04 ^a,b^	4.32 ± 0.55 ^a,b^	0.30 ± 0.06 ^a,b^
HFD + CSE	1.63 ± 0.17 ^a,b^	2.76 ± 0.31 ^a,b^	5.02 ± 0.89 ^a,b^	1.87 ± 0.11 ^a,b^	0.31 ± 0.05 ^a,b^	0.30 ± 0.05 ^a,c^	4.71 ± 0.63 ^a,b^	0.26 ± 0.08 ^a,c^
HFD + M-LB/CS (1:1)	1.17 ± 0.11 ^a,b,d,f^	1.34 ± 0.29 ^a,b,d,f^	2.17 ± 0.49 ^a,b,d,f^	1.26 ± 0.17 ^a,b,d,f^	0.69 ± 0.14 ^a,b,d,f^	0.69 ± 0.10 ^a,b,d,f^	1.51 ± 0.37 ^a,b,d,f^	0.58 ± 0.15 ^a,b,d,f^
HFD + M-LB/CS (1:2)	1.39 ± 0.08 ^a,b,d,g^	1.87 ± 0.37 ^a,b,e,f^	3.89 ± 0.84 ^a,b,e,g^	1.56 ± 0.08 ^a,b,d,f^	0.53 ± 0.11 ^a,b,d,f^	0.51 ± 0.11 ^a,b,d,f^	3.03 ± 0.30 ^a,b,d,f^	0.39 ± 0.07 ^a,b,e,f^
HFD + M-LB/CS (1:4)	1.52 ± 0.16 ^a,b^	2.64 ± 0.39 ^a,b^	4.86 ± 0.83 ^a,b^	1.76 ± 0.22 ^a,b^	0.34 ± 0.08 ^a,b^	0.35 ± 0.08 ^a,b^	4.24 ± 0.68 ^a,b^	0.30 ± 0.07 ^a,b^
HFD + M-LB/CS (1:6)	1.54 ± 0.12 ^a,b^	2.62 ± 0.37 ^a,b^	4.92 ± 0.93 ^a,b^	1.77 ± 0.24 ^a,b^	0.34 ± 0.08 ^a,b^	0.33 ± 0.10 ^a,b^	4.30 ± 0.78 ^a,b^	0.30 ± 0.09 ^a,b^
HFD + M-LB/CS (1:8)	1.55 ± 0.16 ^a,b^	2.74 ± 0.34 ^a,b^	4.98 ± 1.22 ^a,b^	1.86 ± 0.18 ^a,b^	0.32 ± 0.06 ^a,b^	0.34 ± 0.11 ^a,b^	4.30 ± 0.90 ^a,b^	0.30 ± 0.09 ^a,b^
HFD + M-LB/CS (2:1)	1.25 ± 0.09 ^a,b,d,f^	1.52 ± 0.31 ^a,b,d,f^	2.55 ± 0.59 ^a,b,d,f^	1.36 ± 0.11 ^a,b,d,f^	0.63 ± 0.12 ^a,b,d,f^	0.61 ± 0.11 ^a,b,d,f^	2.05 ± 0.59 ^a,b,d,f^	0.51 ± 0.10 ^a,b,d,f^
HFD + M-LB/CS (4:1)	1.34 ± 0.12 ^a,b,d,f^	1.84 ± 0.26 ^a,b,d,f^	3.85 ± 0.53 ^a,b,d,f^	1.51 ± 0.16 ^a,b,e,f^	0.55 ± 0.09 ^a,b,d,f^	0.53 ± 0.09 ^a,b,d,f^	2.81 ± 0.42 ^a,b,d,f^	0.41 ± 0.08 ^a,b,e,f^
HFD + M-LB/CS (6:1)	1.53 ± 0.20 ^a,b^	2.50 ± 0.48 ^a,b^	4.77 ± 1.02 ^a,b^	1.79 ± 0.12 ^a,b^	0.34 ± 0.06 ^a,b^	0.35 ± 0.10 ^a,b^	4.22 ± 0.76 ^a,b^	0.32 ± 0.04 ^a,b^
HFD + M-LB/CS (8:1)	1.54 ± 0.18 ^a,b^	2.45 ± 0.43 ^a,b^	4.80 ± 0.27 ^a,b^	1.76 ± 0.19 ^a,b^	0.36 ± 0.09 ^a,b^	0.37 ± 0.11 ^a,b^	4.32 ± 0.79 ^a,b^	0.32 ± 0.11 ^a,b^

mRNA levels associated with lipogenesis, lipolysis, and adipokines were determined by qPCR analysis. ^a^ *p* < 0.01, vs. NFD; ^b^ *p* < 0.01, ^c^ *p* < 0.05, vs. HFD; ^d^ *p* < 0.01, ^e^ *p* < 0.05, vs. HFD + LBE; ^f^ *p* < 0.01, ^g^ *p* < 0.05, vs. HFD + CSE.

## Data Availability

Data is contained within the article and Supplementary Material.

## Supplementary Materials

The following are available online at https://www.mdpi.com/article/10.3390/antiox10122015/s1, Figure S1: Effect of rosmarinic acid and allantoin on DPPH radical scavenging activity.
