# Supplementary material for: Lemon Balm and Corn Silk Extracts Mitigate High-Fat Diet-Induced Obesity in Mice
Source: Antioxidants (Basel). 2021 Dec 19;10(12):2015. doi: 10.3390/antiox10122015 (PMC8698494; doi:10.3390/antiox10122015)
Supplement: Supplementary file 1 [file antioxidants-10-02015-s001.zip › antioxidants-1488714-supplementary.pdf]

antioxidants

MDPI

Supplementary information

Mixture of Lemon Balm and Corn Silk Extracts Mitigate High-Fat Diet-Induced Obesity in Mice

Il-Je Cho [1,†], Sung-Eon Kim [2,†], Beom-Rak Choi [3], Hye-Rim Park [3], Jeong-Eun Park [3], Seong-Hwa Hong [3], Young-Sam Kwon [2], Won-Seok Oh [4,*] and Sae-Kwang Ku [5,*]

[1] Department of Herbal Prescription, College of Korean Medicine, Daegu Haany University, Gyeongsan, Gyeongsangbuk-do 38610, Korea; skek023@dhu.ac.kr
[2] Department of Veterinary Surgery, College of Veterinary Medicine, Kyungpook National University, Daegu 41566, Korea; veter00@knu.ac.kr (S.-E.K.), kwon@knu.ac.kr (Y.-S.K.)
[3] Nutracore Co., Ltd., Gwanggyo SK Viewlake A-3206, Beobjo-Ro 25, Suwon, Gyeonggi-do 16514, Korea; brchoi@nutracore.co.kr (B.-R.C.), hrpark@nutracore.co.kr (H.-R.P.), jpark@nutracore.co.kr (J.-E.P.), shhong@nutracore.co.kr (S.-H.H.)
[4] Department of Veterinary Internal Medicine, College of Veterinary Medicine, Kyungpook National University, Daegu 41566, Korea
[5] Department of Histology and Anatomy, College of Korean Medicine, Daegu Haany University, Gyeongsan, Gyeongsangbuk-do 38610, Korea
* Correspondence: owsvcs@hanmail.net (W.-S.O.); gucci200@hanmail.net (S.-K.K.); Tel.: +82-53-950-5951 (W.-S.O.); +82-53-819-1549 (S.-K.K.)
† Contributed equally to this work

Supplementary Materials and Methods

2,2-Diphenyl-1-picrylhydrazyl (DPPH) Radical Scavenging Assay

DPPH assay was conducted according to the established method with slight modifications [1]. Briefly, 180 μL of DPPH (150 μM) was reacted with 20 μL of various concentration of rosmarinic acid or allantoin for 30 min in the dark. Equal amount of distilled water was used for vehicle. Optical intensity of the reaction mixture was measured at the wavelength of 517 nm using an EnSpire™ multimode plate reader (PerkinElmer, Waltham, MA, USA).

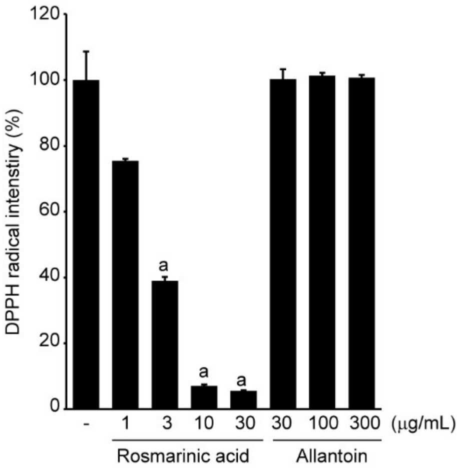

Figure S1. Effect of rosmarinic acid and allantoin on DPPH radical scavenging activity. DPPH radical intensity was calculated as a percentage of the vehicle: [a] $p < 0.01$, versus vehicle.

Supplementary References

1. Choi, B.R.; Cho, I.J.; Jung, S.J.; Kim, J.K.; Park, S.M.; Lee, D.G.; Ku, S.K.; Park, K.M. Lemon balm and dandelion leaf extract synergistically alleviate ethanol-induced hepatotoxicity by enhancing antioxidant and anti-inflammatory activity. *J. Food Bio chem.* **2020**, *44*, e13232. doi: 10.1111/jfbc.13232.
